# Supplementary figures and images for: Blockade of p38 MAPK overcomes AML stem cell line KG1a resistance to 5-Fluorouridine and the impact on miRNA profiling
Source: PLoS One. 2022 May 5;17(5):e0267855. doi: 10.1371/journal.pone.0267855 (PMC9071118; doi:10.1371/journal.pone.0267855)

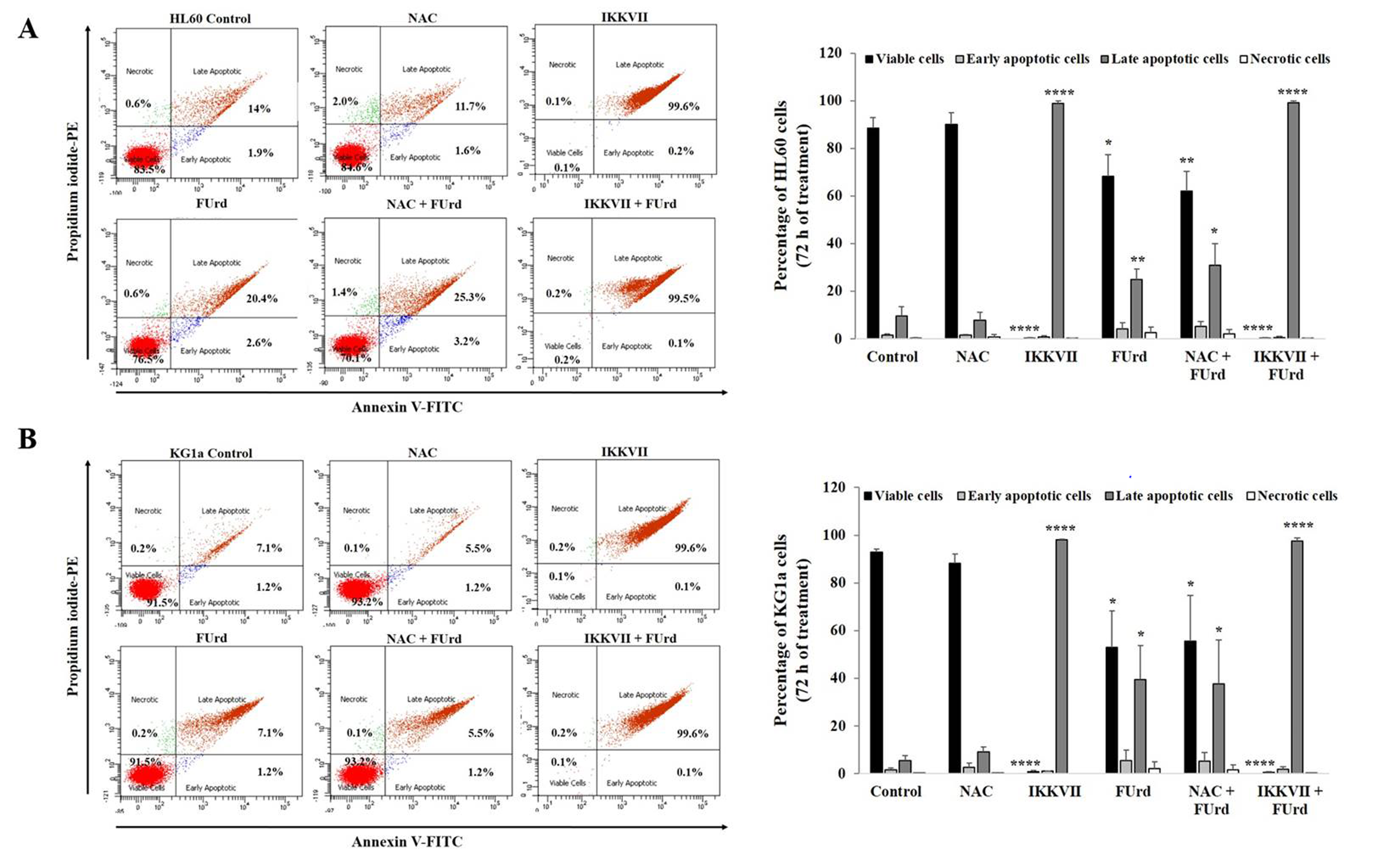

Supplement: S1 Fig — The HL60 (A) and KG1a (B) cells were pre-treated with 5 mM NAC or 20 μM IKKVII for 2 h incubation then followed by the cell treatment with 10 μM of FUrd. After 72 h incubation, viable, (early and late) apoptotic, and necrotic status of the cells were determined using Apoptosis determination kit. Representative cell scatter plots indicating the percentage of cells determined at each status. Bar graphs showing the results presented as mean ± SD, based on three independent experiments. *p < 0.05, **p < 0.01, and ****p < 0.0001 vs. control. (TIF) [file pone.0267855.s001.tif]
